# Metabolomic analysis of fatal hypothermia using ultra-high-performance liquid chromatography‒mass spectrometry

**DOI:** 10.3389/fmolb.2025.1563642

**Published:** 2025-04-16

**Authors:** Jia-Hao Li, Jia-Li Liu, Jian-Wen Song, Wei-Liang Deng, Xin-Zhi Cao, Zhong-Wen Wu, Ding-Hao Chen, Hui Wang, Song Yu, Qi Wang

**Affiliations:** ^1^ Guangzhou Key Laboratory of Forensic Multi-Omics for Precision Identification, School of Forensic Medicine, Southern Medical University, Guangzhou, Guangdong, China; ^2^ Forensic Appraisal Department, Guangdong Provincial Forensic Science of Evidence Materials (Nantian) Engineering Technology Research Center, Shenzhen, Guangdong, China; ^3^ Judicial Appraisal Technology Teaching and Research Office, Guangdong Justice Police Vocational College, Guangzhou, Guangdong, China; ^4^ Department of Pediatric Surgery, Guangzhou Women and Children’s Medical Center, Guangzhou Medical University, National Children’s Medical Center for South Central Region, Guangzhou, Guangdong, China

**Keywords:** fatal hypothermia, metabolomic, UHPLC-MS, identification, forensic medicine

## Abstract

**Introduction:**

The identification of fatal hypothermia remains a significant challenge in forensic medicine. Metabolomics, which reflects the overall changes in endogenous metabolites within an organism, holds substantial value in the exploration of disease mechanisms and the screening of molecular markers.

**Methods:**

Using ultra-high-performance liquid chromatography‒mass spectrometry (UHPLC‒MS), we conducted a metabolomic analysis of serum, heart, lung, and kidney tissues from mice with fatal hypothermia.

**Results:**

A total of 67 metabolites significantly differed across all the tissues, involving pathways such as the TCA cycle, fatty acid oxidation, arginine metabolism, histamine metabolism, and antioxidant-related pathways. Each tissue also displayed unique metabolic alterations. Additionally, we observed significant differences in the metabolomic profiles of kidney tissues from mice with different survival times.

**Conclusion:**

Our findings contribute to elucidate the underlying mechanisms involved and provide a foundation for the forensic identification of markers of fatal hypothermia.

## Graphical Abstract

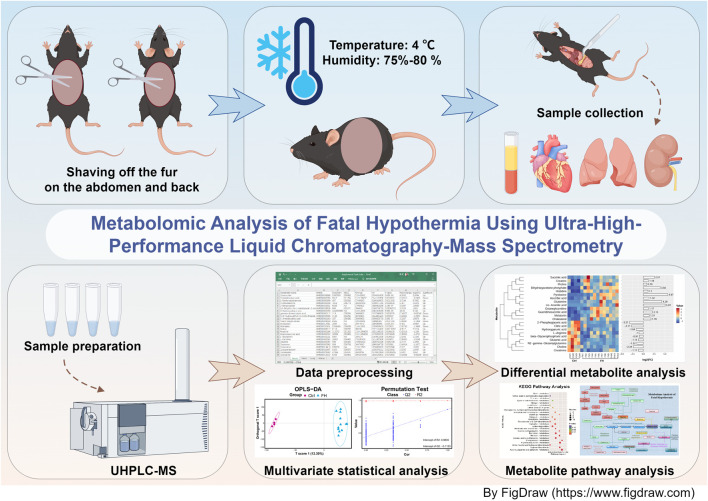

## 1 Introduction

Hypothermia is defined as a core temperature below 35°C and is commonly caused by exposure to cold environments (Palmiere et al., 2014a). In a cold environment, when the body’s heat dissipation exceeds heat production from metabolism or other emergency response pathways, surpassing the physiological limits of body temperature regulation, it can lead to metabolic disorders and physiological dysfunction, ultimately resulting in death. Currently, the forensic identification of fatal hypothermia remains challenging (Rousseau et al., 2018), and the diagnosis relies primarily on nonspecific *postmortem* signs, including frost erythema, Wischnewsky spots (acute gastric erosions), and iliopsoas muscle bleeding (Doberentz and Madea, 2017; Palmiere et al., 2014b; Zatopkova et al., 2017). In the absence of characteristic histopathological features or a detailed medical history, accurately diagnosing lethal hypothermia continues to be a significant challenge in forensic identification.

The rise of metabolomics provides a novel approach for exploring biomarkers, demonstrating potential applications in disease diagnosis and mechanistic studies (Chen et al., 2023; Yu et al., 2024). New technologies such as Ultra-high-performance liquid chromatography‒mass spectrometry (UHPLC-MS), UHPLC-quadrupole time-of-flight mass spectrometry (UHPLC-QTOF-MS), and attenuated total reflection Fourier-transform infrared spectroscopy (ATR-FTIR) have shown value in the forensic identification of fatal hypothermia (Chen et al., 2024; Elmsjo et al., 2024). UHPLC‒MS offers high specificity, high sensitivity, high stability, and the ability to detect multiple analytes simultaneously, making it a valuable tool for screening metabolic profiles in various diseases (Ahmetaj-Shala et al., 2018; Zhang et al., 2019). In forensic science, metabolomics has also played a crucial role in drug identification, determining causes of death, and estimating the time of death (Akcan et al., 2020; Dawidowska et al., 2021; Szeremeta et al., 2021). Previous studies on fatal hypothermia have focused primarily on analyzing changes in catecholamines, cortisol, ketones, and lipids (including acylcarnitines and free fatty acids) in tissues and bodily fluids such as serum, urine, vitreous humor, and brown adipose tissue (Hervet et al., 2016; Hirvonen and Huttunen, 1995; Kaija et al., 2014; Lee et al., 1997; Lin et al., 2019a; Palmiere et al., 2013; Palmiere et al., 2014a; Rousseau et al., 2021). Urinary catecholamines and blood ketones have been utilized to support the diagnosis of fatal hypothermia (Pakanen et al., 2011; Palmiere and Mangin, 2012). Studies have also focused on the mRNA and protein changes associated with fatal hypothermia (Du SH et al., 2017; Umehara et al., 2019; Wang et al., 2012; Wang et al., 2013; Wang et al., 2011). However, research on metabolic alterations and biomarkers associated with fatal hypothermia remains limited (Elmsjo et al., 2024; Rousseau et al., 2021; Rousseau et al., 2019), with a primary focus on bodily fluids. Additional research is needed to elucidate metabolic changes in tissues and identify tissue-specific biomarkers.

This study established a fatal hypothermia mouse model simulating human freezing to death in cold environments. We performed UHPLC‒MS metabolomic analysis on serum, heart, lung, and kidney tissues. Our findings reveal new insights into the tissue-specific metabolic changes induced by fatal hypothermia and provide a foundation for screening promising biomarkers.

## 2 Materials and methods

### 2.1 Animals and treatment

Ten-week-old male C57BL/6 mice were used in this study. A total of sixteen mice used in this experiment were kept in the same specific pathogen-free (SPF) environment (indoor temperature: 20°C–26°C, humidity: 40%–70%) and feeding conditions, with free access to water and food. The experimental mice in each group were homogeneous in terms of breed and feeding. All animal care and experimental protocols were approved by the Ethics Committee of Southern Medical University (SMUL202405019).

After a 3-day acclimatization period, the experiment was initiated. The mice were randomly divided into a fatal hypothermia group (FH group, n = 12) and a control group (Ctrl group, n = 4). After the fur on the back and abdomen was shaved (to simulate the condition of thin clothing in humans), the mice in the FH group were placed alone in a damp cage without bedding material, water or food and then transferred to a cold room maintained at 4°C with a humidity of 75%–80% (to simulate a wet and cold environment typical during low-temperature exposure) until death. This model draws on previous research to simulate fatal hypothermia in humans (Lin et al., 2019b). The survival time of each mouse was recorded (Supplemental Figures S1A, B). During the experiment, we made every effort to reduce the pain of the mice. The experimental endpoint was defined as the point at which the mouse respiration and heart activity became so faint that they were no longer detectable by visual inspection or palpation. Upon reaching the endpoint of our experiment, anesthesia was induced in mice using an intraperitoneal injection of 0.3% pentobarbital sodium at a dose of 0.1 mL/10 g (3 mg/kg) body weight. Once deep anesthesia was confirmed by the absence of a toe-pinch reflex, the mice were sacrificed by cervical dislocation. Immediately following euthanasia, tissue samples including heart, lung, and kidney were promptly collected. These tissues were snap-frozen in liquid nitrogen and stored at −80°C. Blood samples were collected, allowed to clot at room temperature for 1 h, and then centrifuged at 4°C for 10 min at 3,000 rpm to obtain serum. After fasting for 3.5 h, the control mice were euthanized directly after the back and belly fur were shaved. Mouse serum, heart, lung, and kidney tissues were collected as described above for subsequent experiments.

### 2.2 Ultra-high-performance liquid chromatography‒mass spectrometry (UHPLC‒MS)

#### 2.2.1 Sample preparation

For plasma nontargeted metabolomics, 50 μL of plasma was mixed with 200 μL of methanol. The mixture was vortexed for 15 min and centrifuged at 4°C and 13,000 rpm for 20 min. The supernatant was transferred to a new tube, concentrated to dryness under vacuum, and stored at −80°C until analysis. Before analysis, the dried samples were reconstituted in 100 μL of acetonitrile (1:1, v/v). The reconstituted samples were centrifuged at 12,000 rpm for 15 min, and 10 μL of the supernatant was injected for UHPLC-MS analysis.

For nontargeted metabolomics, 80 μg of tissue was homogenized in 800 μL of methanol. The mixture was vortexed for 15 min and centrifuged at 4°C and 13,000 rpm for 20 min. The supernatant was transferred to a new tube, concentrated to dryness under vacuum, and stored at −80°C until analysis. The subsequent steps were identical to those for the plasma samples.

All the samples were processed under uniform conditions to avoid biases introduced during sample preparation.

#### 2.2.2 Ultra-high performance liquid chromatography (UHPLC)

The prepared samples were reconstituted, diluted, and analyzed via an Ultimate 3000 UHPLC system coupled with a Q Exactive MS (Thermo Scientific). Chromatographic separation was performed on a Waters XBridge BEH Amide column (100 × 2.1 mm, 2.5 μm) at an injection temperature of 4°C and an injection volume of 5 μL. The mobile phase consisted of two solvents: A (5 mM ammonium acetate in 5% acetonitrile and 95% water) and B (acetonitrile). The gradient elution conditions were as follows: 0–2 min, 95% B; 2–15 min, 95% B to 50% B; 15–18 min, 50% B; 18–19 min, 50% B to 95% B; and 19–23 min, 95% B. The flow rate was 0.35 mL/min.

#### 2.2.3 Mass spectrometry detection

Nontargeted mass spectrometry detection was performed via a Q Exactive MS instrument equipped with a HESI ion source (Thermo Scientific). Data were acquired in both positive and negative ion modes. The ion source parameters were set as follows: ion source temperature, 320°C; desolvation gas temperature, 300°C; sheath gas, 40 arbitrary units (arb); and auxiliary gas, 10 arb. The capillary voltage was +3.3 kV in positive mode and −3.0 kV in negative mode, with a cone voltage of 0 V. The mass range was 60–800 m/z, and the data were collected in data-dependent acquisition (DDA) mode. MS1 scans were performed at a resolution of 35,000, and MS/MS scans were performed at a resolution of 17,500. The automatic gain control (AGC) targets were set to 5e6 (maximum injection time, 100 ms) for MS1 and 2e5 (maximum injection time, 64 ms) for MS/MS. Dynamic exclusion was set to 8 s, and the normalized collision energy (NCE) settings were 15, 30, and 45.

To ensure the stability and reliability of the data, quality control (QC) samples were prepared by mixing all the serum samples. QC samples were run before and after the sequence and every 15 samples within the sequence. PLS-DA analysis of the QC samples revealed good clustering among the QC samples (Supplemental Figures S1C–F).

#### 2.2.4 Data processing

The raw data were processed via MS-DIAL software to generate Excel tables containing m/z values, retention times, peak areas, and MS/MS fragmentation patterns. Missing data were imputed with minimal values. Compound identification was based on MS1 and MS/MS spectra matched against the MassBank database (https://massbank.eu/), with MS1 and MS/MS search thresholds set to 0.01 Da and 0.05 Da, respectively. The data were further analyzed via the MetaboAnalyst website (https://www.metaboanalyst.ca/MetaboAnalyst/) to perform normalization, standardization, and log transformation for subsequent statistical analysis.

### 2.3 Statistical analysis

Basing on MetaX (version 2.0.0), orthogonal partial least squares discriminant analysis (OPLS-DA) and partial least squares discriminant analysis (PLS-DA) were employed to identify metabolic differences between the fatal hypothermia group (FH group) and the control group (Ctrl group) in various tissues. The OPLS-DA and PLS-DA models were validated via seven-fold cross-validation and 200-fold response permutation tests. The variable importance in projection (VIP) values were used to evaluate the contribution of each metabolite to the classification. Metabolites with a VIP ≥1 and P value <0.01 were selected as differentially expressed metabolites. A summary of the differentially expressed metabolites is presented in Supplemental Table S2. KEGG pathway analysis was performed via the MetaboAnalyst website (https://www.metaboanalyst.ca/MetaboAnalyst/). The fold change (FC) represents the ratio of metabolite expression levels in the FH group to those in the Ctrl group. A positive log2 (FC) value indicates upregulation, whereas a negative log2 (FC) value indicates downregulation. Differentially expressed metabolites were standardized via the Z score, and a heatmap was generated to visualize the results via the OmicStudio platform (https://www.omicstudio.cn/home).

## 3 Results

### 3.1 Serum metabolomic changes induced by fatal hypothermia

**FIGURE 1:**
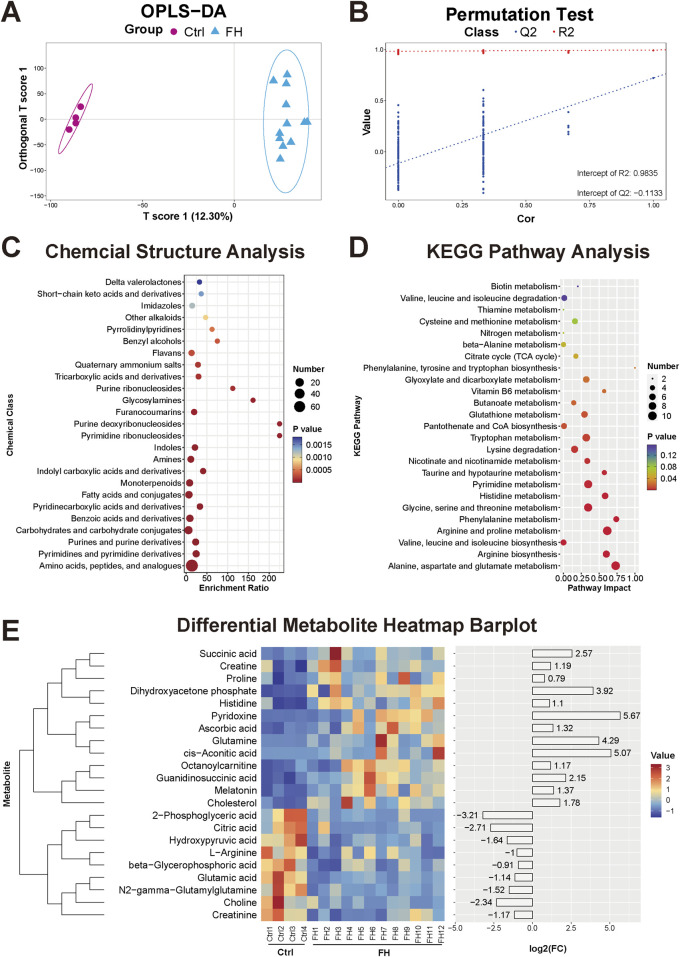
Serum metabolomic changes induced by fatal hypothermia **(A)** Serum metabolome OPLS-DA score plots and **(B)** permutation test results. **(C)** Chemical structure enrichment analysis diagram of the differentially expressed metabolites, showing the top 25 enrichment results. **(D)** KEGG pathway analysis of the differentially expressed metabolites. The color of the point reflects the P value, while its size denotes the number of enriched different metabolites. **(E)** Heatmap of differentially expressed metabolites. Red represents upregulated metabolite expression, and blue represents downregulated metabolite expression compared with the control group. The log2 (FC) shows the difference multiples, with positive numbers indicating up-modulation and negative numbers indicating down-modulation.

Nontargeted metabolomics analysis via UHPLC‒MS was performed on the serum of both the control group and the FH group. The OPLS-DA model clearly revealed separation between the two groups (Figure 1A). Validation of the OPLS-DA model via permutation tests yielded R^2^X = 0.992, R^2^Y = 0.9835, Q^2^X = 0.722, and Q^2^Y = −0.1133, indicating good explanatory and predictive capabilities (Figure 1B). A total of 388 differentially expressed metabolites were identified in the serum. Further classification revealed that these metabolites were primarily amino acids and peptide compounds, purines and pyrimidine compounds, carbohydrates, fatty acids and their derivatives, and amine compounds (Figure 1C). Subsequent KEGG functional enrichment analysis of the differentially expressed metabolites revealed significant involvement in the tricarboxylic acid (TCA) cycle, amino acid biosynthesis and degradation, arginine biosynthesis, histidine metabolism, nitrogen metabolism, glutathione metabolism, pyrimidine metabolism, and taurine metabolism (Figure 1D). Heatmaps were generated to visualize representative differentially expressed metabolites (Figure 1E).

### 3.2 Heart tissue metabolomic changes induced by fatal hypothermia

**FIGURE 2:**
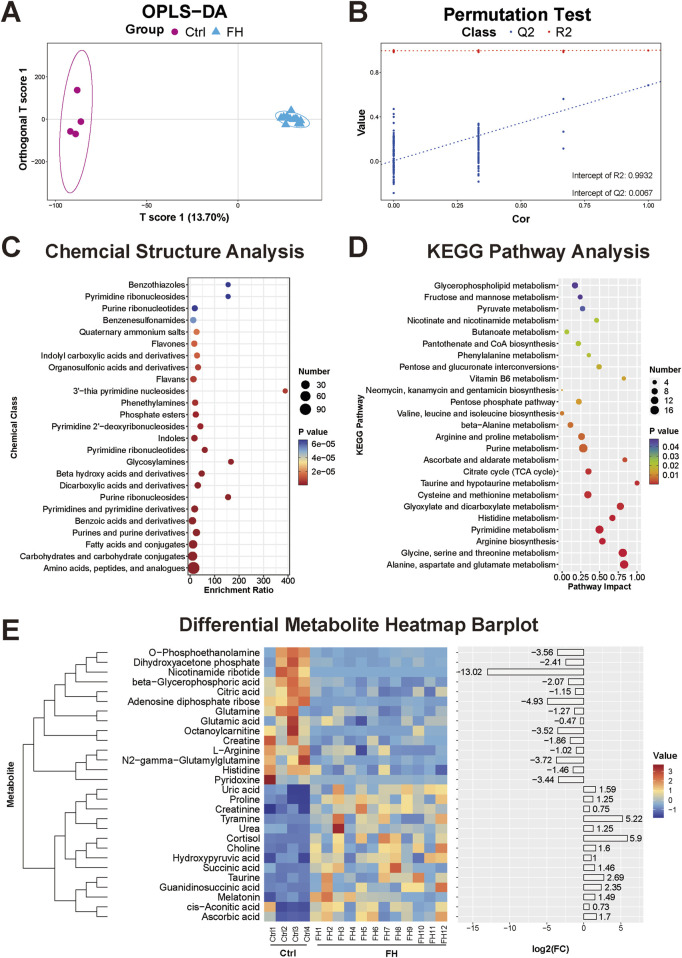
Heart tissue metabolome changes induced by fatal hypothermia **(A)** The heart tissue metabolome OPLS-DA score plots and **(B)** permutation test results. **(C)** Chemical structure enrichment analysis diagram of the differentially expressed metabolites, showing the top 25 enrichment results. **(D)** KEGG pathway analysis of the differentially expressed metabolites. The color of the point reflects the P value, while its size denotes the number of enriched different metabolites. **(E)** Heatmap of differentially expressed metabolites. Red represents upregulated metabolite expression, and blue represents downregulated metabolite expression compared with the control group. The log2 (FC) shows the difference multiples, with positive numbers indicating up-modulation and negative numbers indicating down-modulation.

The OPLS-DA model for the metabolomics analysis of heart tissue also showed distinct clustering of samples from the FH group and the control group, with a clear separation between the two groups (Figure 2A). The stability and reliability of the OPLS-DA model were confirmed through permutation tests, which yielded R^2^X = 0.996, R^2^Y = 0.9932, Q^2^X = 0.684, and Q^2^Y = 0.0067 (Figure 2B). A total of 566 differentially expressed metabolites were identified in heart tissue. These metabolites were primarily categorized into amino acids and peptides, carbohydrates and carbohydrate conjugates, fatty acids and conjugates, purines and pyrimidine derivatives, dicarboxylic acids and derivatives, and beta hydroxy acids and derivatives (Figure 2C). Many of these metabolites are closely related to energy metabolism. KEGG functional enrichment analysis revealed the significant involvement of several energy metabolism pathways, including glycerophospholipid metabolism, fructose and mannose metabolism, pyruvate metabolism, the pentose phosphate pathway, and the tricarboxylic acid (TCA) cycle (Figure 2D). Heatmaps were constructed to display representative differentially expressed metabolites (Figure 2E).

### 3.3 Lung tissue metabolomic changes induced by fatal hypothermia

**FIGURE 3:**
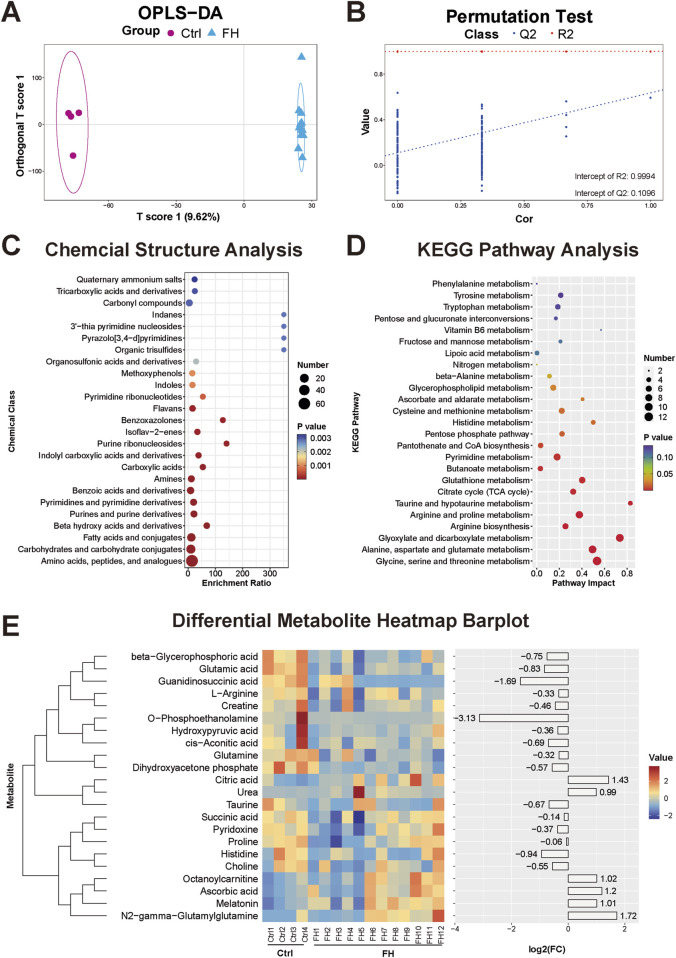
Lung tissue metabolomic changes induced by fatal hypothermia **(A)** Lung tissue metabolome OPLS-DA score plots and **(B)** permutation test results. **(C)** Chemical structure enrichment analysis diagram of the differentially expressed metabolites, showing the top 25 enrichment results. **(D)** KEGG pathway analysis of the differentially expressed metabolites. The color of the point reflects the P value, while its size denotes the number of enriched different metabolites. **(E)** Heatmap of differentially expressed metabolites. Red represents upregulated metabolite expression, and blue represents downregulated metabolite expression compared with the control group. The log2 (FC) shows the difference multiples, with positive numbers indicating up-modulation and negative numbers indicating down-modulation.

In the lung tissue, OPLS-DA revealed distinct clustering of samples from the FH group, clearly separating them from the control group (Figure 3A). These clustering patterns indicate significant alterations in the lung metabolome due to fatal hypothermia. Permutation tests verified the stability of the OPLS-DA model, demonstrating its ability to explain and predict differences in the lung metabolome. The permutation test results were R^2^X = 0.999, R^2^Y = 0.9994, Q^2^X = 0.594, and Q^2^Y = 0.1096 (Figure 3B). Compared with those in the control group, 312 differentially expressed metabolites were identified in the lung tissue of the FH group. The types of metabolites were similar to those found in serum and heart tissue, primarily consisting of amino acids and peptides, carbohydrates and their conjugates, and fatty acids and their derivatives (Figure 3C). KEGG functional enrichment analysis highlighted several key metabolic pathways, including the tricarboxylic acid (TCA) cycle, arginine biosynthesis and metabolism, histidine metabolism, ester acid metabolism, glycerophospholipid metabolism, glutathione metabolism, and β-alanine metabolism (Figure 3D). Representative differentially expressed metabolites were visualized via heatmaps (Figure 3E).

### 3.4 Kidney tissue metabolomic changes induced by fatal hypothermia

**FIGURE 4:**
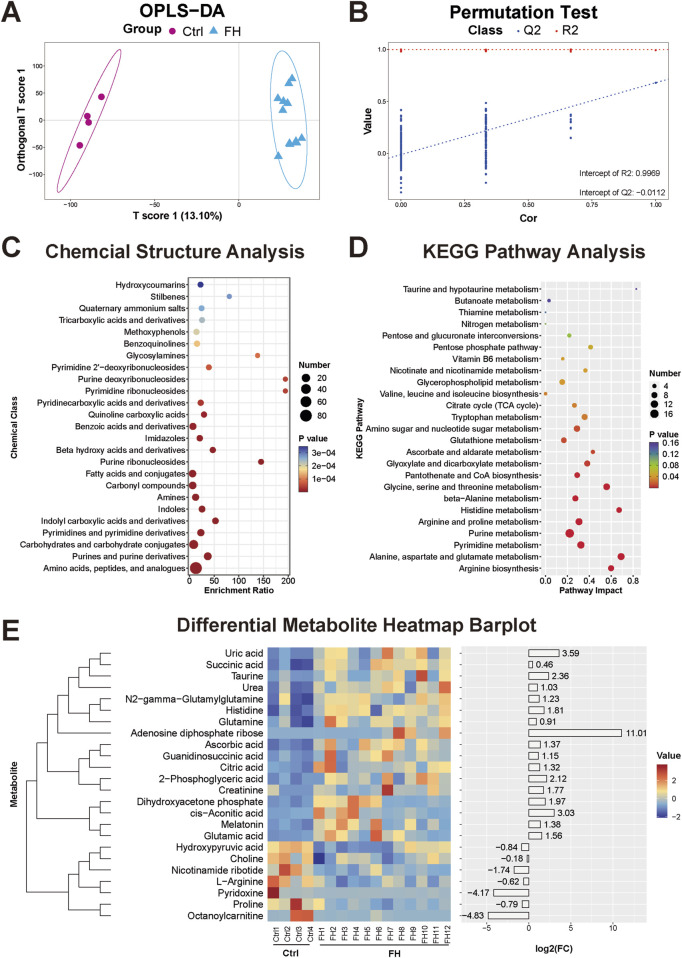
Kidney tissue metabolomic changes induced by fatal hypothermia **(A)** Kidney tissue metabolome OPLS-DA score plots and **(B)** permutation test results. **(C)** Chemical structure enrichment analysis diagram of the differentially expressed metabolites and the top 25 enrichment results. **(D)** KEGG pathway analysis of the differentially expressed metabolites. The color of the point reflects the P value, while its size denotes the number of enriched different metabolites. **(E)** Heatmap of differentially expressed metabolites. Red represents upregulated metabolite expression, and blue represents downregulated metabolite expression compared with the control group. The log2 (FC) shows the difference multiples, with positive numbers indicating up-modulation and negative numbers indicating down-modulation.

The kidney metabolism of the FH group was significantly different from that of the control group, as evidenced by the clear separation between the two groups in OPLS-DA (Figure 4A). The stability and reliability of the OPLS-DA model were confirmed through permutation tests, with R^2^X = 0.993, R^2^Y = 0.9969, Q^2^X = 0.68, and Q^2^Y = −0.0112 (Figure 4B). A total of 456 differentially expressed metabolites were identified, primarily consisting of amino acids, peptides and analogues, purines and purine derivatives, carbohydrates and carbohydrate conjugates, amines, and indolyl carboxylic acids and their derivatives (Figure 4C). These differentially expressed metabolites are likely involved in several key metabolic pathways, including arginine biosynthesis and metabolism, purine and pyrimidine metabolism, histidine metabolism, B-alanine metabolism, glyoxylate and dicarboxylate metabolism, and the tricarboxylic acid (TCA) cycle (Figure 4D). Some of the metabolites that may have potential roles and application value are shown in the heatmap (Figure 4E).

**FIGURE 5:**
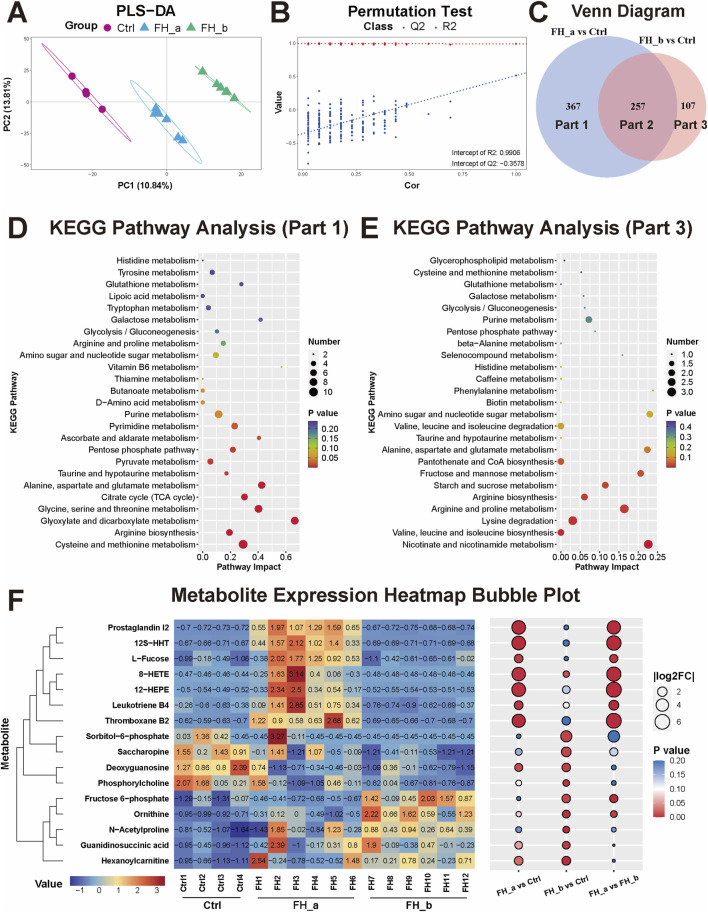
Differential metabolomic profiles in the FH_a and FH_b groups **(A)** The kidney tissue metabolome PLS‒DA score plots and **(B)** permutation test results among the FH_a, FH_b and Ctrl groups. **(C)** Venn analysis of differences in metabolites between the two groups and the control group. **(D)** KEGG pathway analysis of differentially expressed metabolites unique to the FH_a group (Part 1) and **(E)** FH_b group (Part 3). The color of the point reflects the P value, while its size denotes the number of enriched different metabolites. **(F)** Heatmap of differentially expressed metabolites. The colors represent the value. The numbers in the heatmap represent the standardized levels of expression. In the bubble diagram on the right, the size of the bubble represents |log2FC|, and the colors represent the P value.

Notably, we observed that there were two distinct clusters within the FH group. By tracking the time elapsed from freezing to death in the mice, we found that these clusters were closely related to the duration of freezing. To further investigate this relationship, we divided the mice into two subgroups on the basis of the time elapsed from freezing to death: the rapid freezing group (time elapsed from freezing to death ≤180 min, designated FH_a) and the slow freezing group (time elapsed from freezing to death >180 min, designated FH_b). We then conducted a detailed analysis of kidney tissue metabolomics for these subgroups. The PLS-DA results revealed that the FH_a group, the FH_b group, and the control group presented significantly different metabolite patterns (Figure 5A). The stability and reliability of the model were verified via permutation tests, with results of R^2^X = 0.9577, R^2^Y = 0.9906, Q^2^X = 0.9356, and Q^2^Y = −0.3578, indicating the model’s strong ability to explain and predict metabolite differences (Figure 5B). Venn analysis revealed that there were 257 common distinct metabolites between the FH_a and FH_b groups, with 367 unique metabolites in the FH_a group and 107 in the FH_b group (Figure 5C). KEGG pathway analysis was performed to explore the potential functions of these metabolites (Figures 5D, E). Pathways such as cysteine and methionine metabolism, D-amino acid metabolism, thiamine metabolism, and vitamin B6 metabolism were significantly enriched in the unique metabolites of the FH_a group, which may help explain the mechanisms underlying the short elapsed time from freezing to death. Representative differentially expressed metabolites were visualized via heatmaps (Figure 5F).

### 3.5 Integrated metabolomic analysis of all tissues

**FIGURE 6:**
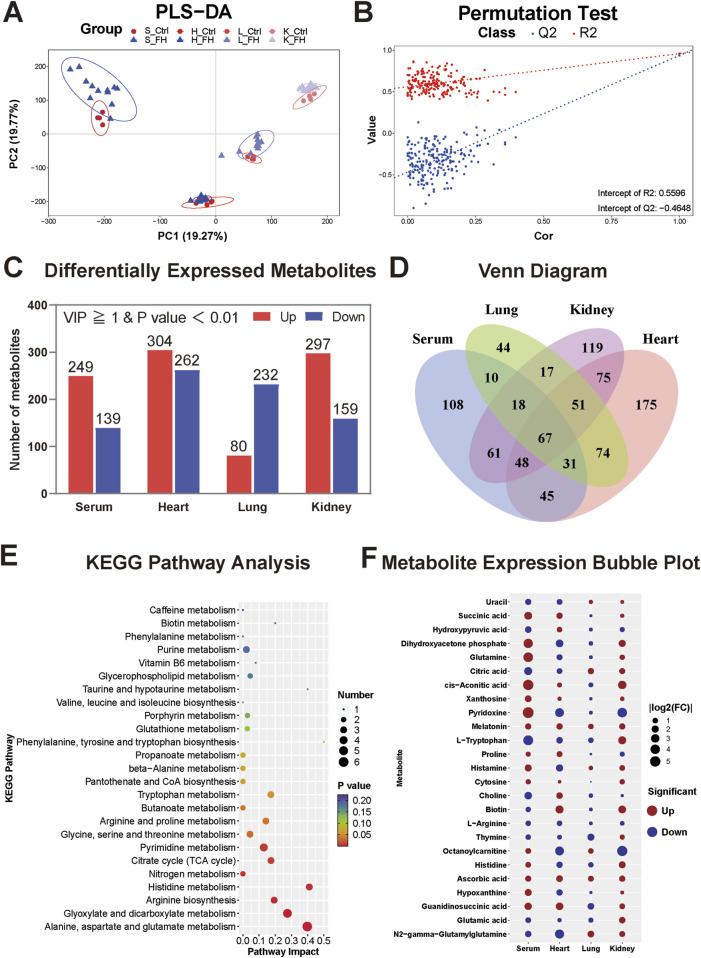
Integrated metabolomic analysis of all tissues **(A)** PLS-DA score plots and **(B)** permutation test results for all tissues. **(C)** The number of differentially expressed metabolites in each tissue compared with that in the control group. **(D)** Results of the Venn analysis of different metabolites. Sixty-seven metabolites were significantly different across all the tissues. **(E)** KEGG pathway analysis of 67 common differentially expressed metabolites. The color of the point reflects the P value, while its size denotes the number of enriched different metabolites. **(F)** Bubble heatmap representing some of the common differentially expressed metabolites. The size of the bubble represents |log2FC|, and the colors denote the direction of change: red represents upregulation, and blue represents downregulation.

Finally, we performed a pooled analysis of all the tissues to explore the common changes in the tissues caused by fatal hypothermia. The PLS-DA results revealed that each organ presented unique metabolic characteristics and that fatal hypothermia induced significant alterations in the metabolome (Figure 6A). The PLS-DA model was validated via a permutation test, with R^2^X = 0.99, R^2^Y = 0.5596, Q^2^X = 0.5138, and Q^2^Y = −0.4648, confirming the model’s stability and reliability (Figure 6B). Venn analysis revealed that 67 metabolites presented significant differences across all the tissues (Figures 6C, D), suggesting that these metabolites may be closely associated with fatal hypothermia. KEGG pathway analysis revealed that several metabolic pathways were significantly enriched in these metabolites, including arginine biosynthesis, histidine metabolism, nitrogen metabolism, the tricarboxylic acid cycle (TCA cycle), glyoxylate and dicarboxylate metabolism, purine and pyrimidine metabolism, glycerophospholipid metabolism, taurine and hypotaurine metabolism, glutathione metabolism, and vitamin B6 metabolism (Figure 6E). These pathways are closely related to energy metabolism, antioxidant defense, and histamine metabolism, highlighting their potential roles in the pathophysiology of fatal hypothermia. The bubble heatmap shows the specific changes of common differential metabolites among different tissues (Figure 6F).

## 4 Discussion

Fatal hypothermia affects multiple organs differently. Using UHPLC-MS analysis, we identified 388, 566, 312, and 456 differentially expressed metabolites in the serum, heart, lung, and kidney tissues, respectively. Among these, 67 metabolites were significantly different across all the tissues. Below, we discuss both the common differentially expressed metabolites and those unique to each tissue group.

### 4.1 Common metabolite analysis

Our multi-tissue metabolomic analysis revealed consistent alterations in energy metabolism, antioxidant responses, and vasoregulatory pathways during fatal hypothermia. Key TCA cycle intermediates including succinate, citrate, and cis-aconitate, along with glycolytic derivatives such as dihydroxyacetone phosphate and hydroxypropanoate, exhibited significant changes. These findings were supported by elevated serum octanoylcarnitine levels, which are indicative of activated β-oxidation (Yu et al., 2002). These findings align with previous reports of cold-induced metabolic rewiring (Elmsjo et al., 2024; Rousseau et al., 2021). The enrichment of the pyrimidine/purine pathway likely reflects ATP depletion and nucleic acid degradation under cold stress (Slor et al., 1977). Notably, antioxidant metabolites, including ascorbic acid, pyridoxine, taurine, and melatonin, are universally depleted, which is consistent with cold-induced oxidative stress (Alva et al., 2013; Ibrahim et al., 2022). Concurrent reductions in glutamate/glutamine - glutathione precursors - suggest compromised antioxidant capacity in cardiopulmonary tissues. The tissue-specific histamine elevation observed in serum, lung, and kidney contrasts with prior urinary analyses (Hirvonen, 1976), whereas pan-tissue arginine depletion aligns with increased serum arginase levels in hypothermia fatalities (Rousseau et al., 2019), given the dual role of arginine in nitric oxide-mediated vasodilation (Menzel et al., 2018). and ischemic protection (Amrani et al., 1997; STOWE et al., 1997), its depletion may impair hemodynamic adaptation. Furthermore, studies have demonstrated that arginine stimulation in hypoxic environments leads to a significant increase in plasma glucose levels (Gehrand et al., 2016), indicating that arginine plays a crucial role in physiological regulation under adverse conditions. In our experiments, arginine levels were significantly reduced across all tissues, suggesting that this important regulatory mechanism may be inhibited or depleted under lethal hypothermic conditions. This finding underscores the critical role of arginine in response to extreme environmental stress and suggests that its depletion could contribute to the physiological dysregulation observed in fatal hypothermia. These coordinated changes in histidine/arginine metabolism suggest their involvement in thermoregulatory failure, though causal relationships require mechanistic validation.

### 4.2 Serum metabolite analysis

In the serum, we identified many long-chain fatty acids associated with β-oxidation, such as arachidonic acid, α-linolenic acid, and palmitic acid, along with their carnitine esters, including decanoyl-carnitine, dodecanoyl-carnitine, octanoyl-carnitine, palmitoyl-carnitine, and oleoyl-carnitine. Previous studies have reported that changes in fatty acids and acylcarnitines in the blood lead to fatal hypothermia (Gong et al., 2023; Lin et al., 2019b; Palmiere et al., 2013; Rousseau et al., 2019). Elevated levels of palmitic acid, stearic acid, and oleic acid have been proposed as potential markers of fatal hypothermia (Banka et al., 2014). Our results are consistent with these findings, which revealed increased levels of palmitic acid in the serum. Additionally, we observed a significant increase in amino acid levels in the serum (Gong et al., 2023; Rousseau et al., 2021), suggesting active protein catabolism due to fatal hypothermia. Overall, the increased levels of lipids and amino acids in the serum indicate excessive catabolism of fatty acids and proteins in the body during lethal hypothermia.

### 4.3 Cardiac metabolite analysis

Cardiac-specific metabolic signatures revealed dual-phase adaptive failure. Depleted phosphagen reserves (creatine/ADP) paralleled reduced pericardial CK-MB levels (Wang et al., 2011), indicating that compromised high-energy phosphate cycling is essential for contractile function (Balestrino, 2021; Ingwall, 2009). Concurrently, diminished cardiac lactate - contrary to typical hypoxic glycolysis patterns, suggested the suppression of anaerobic compensation. These energy deficits coincided with neuroendocrine activation, as evidenced by elevated tyramine (sympathetic agonist) and myocardial cortisol, extending previous serum/urinary findings (Banka et al., 2013; Palmiere et al., 2013). The paradoxical coexistence of metabolic exhaustion and catecholaminergic stimulation suggests a failed compensatory escalation preceding terminal cardiac dysfunction. Specifically, the observed reduction in creatine and ADP levels, coupled with decreased lactate, indicates an inability to maintain adequate ATP production under hypoxic conditions. Moreover, increased tyramine and cortisol levels reflect attempts by the body to compensate through heightened sympathetic nervous system activity and glucocorticoid-mediated stress responses.

### 4.4 Lung metabolite analysis

Unlike other tissues, we did not identify many additional metabolites in the lung tissue that could be considered highly specific. Cold exposure leads to a series of changes in the respiratory system, beginning with an initial increase in the respiratory rate and progressing to shallower and slower breathing as hypothermia advances (Schweitzer et al., 2014). The content of beta-sheet protein conformational structures was found to be significantly elevated in the pulmonary edema fluid of fatal hypothermia patients but was not detected in this study (Lin et al., 2020). The analysis of lung gas content or electrolyte changes by imaging or biochemical tests may provide more insights into changes in lung respiration and gas exchange during the process of fatal hypothermia (Palmiere and Mangin, 2012; Sogawa et al., 2014).

### 4.5 Renal metabolite analysis

The renal metabolic response exhibited temporal progression patterns correlated with the duration of cold exposure. In the acute-phase kidneys from the FH_a group, we observed elevated levels of 12-HEPE, a cold-adapted lipid known to increase adipocyte glucose uptake (Leiria et al., 2019), alongside oxidative mediators such as 8-HETE and inflammatory mediators such as 12S-HHT, TXB2, PGI2, and LTB4. These arachidonic acid (AA)-derived mediators are established drivers of renal inflammation in acute kidney injury (Li et al., 2018; Remuzzi et al., 1992). In contrast, in the FH_b group, we observed a significant increase in various intermediate metabolites, including ornithine (a urea cycle intermediate), fructose 6-phosphate (a glycolysis intermediate), hexanoylcarnitine (a fatty acid metabolism intermediate), N-acetylproline (an intermediate in glutathione metabolism), and guanidinosuccinic acid (an intermediate in arginine metabolism). The accumulation of these intermediate metabolites suggests disruption and dysfunction of renal metabolic pathways, indicating a progressive decompensation that has surpassed adaptive thresholds.

There are several limitations to this study. In terms of the number of animals, the imbalanced sample sizes may increase the false positive rate in differentially abundant metabolite screening. Future studies with larger and more balanced sample sizes are needed to further validate our findings. Age and sex have significant effects on the metabolome. In our study, we selected adult male mice aged 10 weeks to minimize potential biases introduced by estrogen fluctuations and menstrual cycles in females, as well as to avoid the confounding factor of accidental death. However, studies involving females and other age groups are crucial for obtaining a comprehensive understanding of the metabolome. Catecholamines play a crucial role in lethal hypothermia. However, identifying the hypothermia-specific mechanisms underlying freezing to death remains an area for further exploration. Future studies could leverage more advanced techniques or models to better understand and distinguish the specific contributions of various stress responses during lethal hypothermia. Finally, our experiments were conducted in mice, which inherently limits the direct application and translation of our findings to the field of forensic medicine. While murine models provide valuable insights into the metabolic changes associated with lethal hypothermia, the extrapolation of these results to human scenarios requires cautious interpretation. Further research, including studies with human samples, is essential to validate our findings and identify robust molecular markers for the diagnosis of fatal hypothermia.

## 5 Conclusion

**FIGURE 7:**
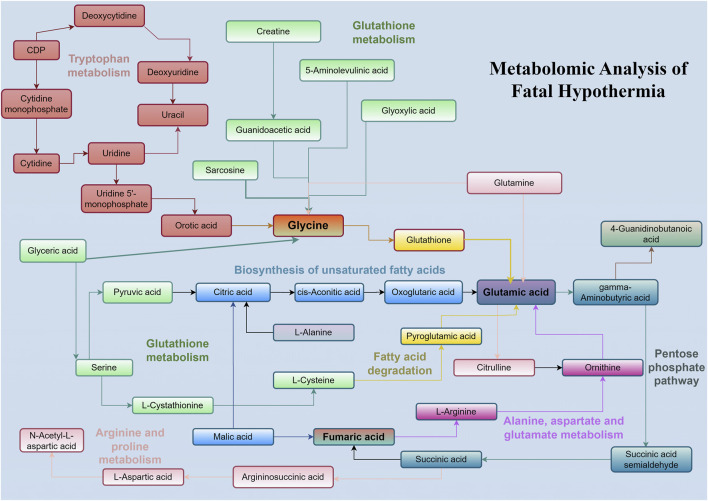
Schematic diagram of metabolic pathways involving differentially expressed metabolites. Differentially expressed metabolites that may be associated with fatal hypothermia and the metabolic pathways involved, including alanine, aspartate and glutamate metabolism, arginine and proline metabolism, fatty acid degradation, pentose phosphate pathway, glutathione metabolism, and tryptophan metabolism. The metabolites shown here are all derived from the 67 common differentially abundant metabolites identified in Figure 5D, which were screened on the basis of the criteria of VIP ≥1 and P value <0.01, indicating significant differences across all tissues. By Figdraw (https://www.figdraw.com).

Owing to the lack of characteristic lesions and diagnostic criteria, identifying deaths caused by fatal hypothermia remains a significant challenge in forensic medicine. New technologies such as UHPLC-MS, UHPLC-QTOF-MS, and ATR-FTIR have shown value in the forensic identification of fatal hypothermia and hold the potential to improve the accuracy of forensic assessments in cases involving fatal hypothermia. Our study utilized UHPLC-MS to analyze the metabolomes of serum, heart, lung, and kidney tissues. This investigation revealed that lethal hypothermia can lead to an energy crisis characterized by generalized tissue depletion of TCA intermediates (such as succinic acid and citric acid) and collapse of cardiac phosphates (including creatine and ADP), resulting in reduced ATP synthesis. Additionally, we observed an oxidative damage cascade characterized by the consumption of general antioxidants (such as ascorbic acid and taurine), and vascular regulatory failure indicated by a systemic decrease in arginine and an increase in histamine. We summarize the differentially expressed metabolites potentially associated with fatal hypothermia and the metabolic pathways involved (Figure 7). These findings not only elucidate the potential mechanisms of fatal hypothermia through the examination of related metabolites and their pathways but also provide empirical evidence for screening metabolites linked to fatal hypothermia. By leveraging these advanced analytical techniques, our study aims to enhance the understanding and technological capabilities of forensic medicine, ultimately contributing to more accurate diagnoses in cases of fatal hypothermia.

## Data Availability

The original contributions presented in the study are included in the article/Supplementary Material, further inquiries can be directed to the corresponding authors.

## Supplementary material

SUPPLEMENTARY TABLE S1 Raw date processed via MS-DIAL software, containing m/z values, retention times, and relative expression of metabolites.

SUPPLEMENTARY TABLE S2 Summary table of differential metabolites.

SUPPLEMENTARY FIGURE S1
**(A)** Survival curve of the fatal hypothermia mouse model. **(B)** Survival time of fatal hypothermia mice. **(C)** PLS-DA results for the serum samples, including the control group, FH group and QC samples. **(D)** PLS-DA results for heart tissue. **(E)** PLS-DA results for lung tissue. **(F)** PLS-DA results for kidney tissue.
