# Supplementary figures and images for: Metabolomic analysis of fatal hypothermia using ultra-high-performance liquid chromatography‒mass spectrometry
Source: Front Mol Biosci. 2025 Apr 16;12:1563642. doi: 10.3389/fmolb.2025.1563642 (PMC12040645; doi:10.3389/fmolb.2025.1563642)

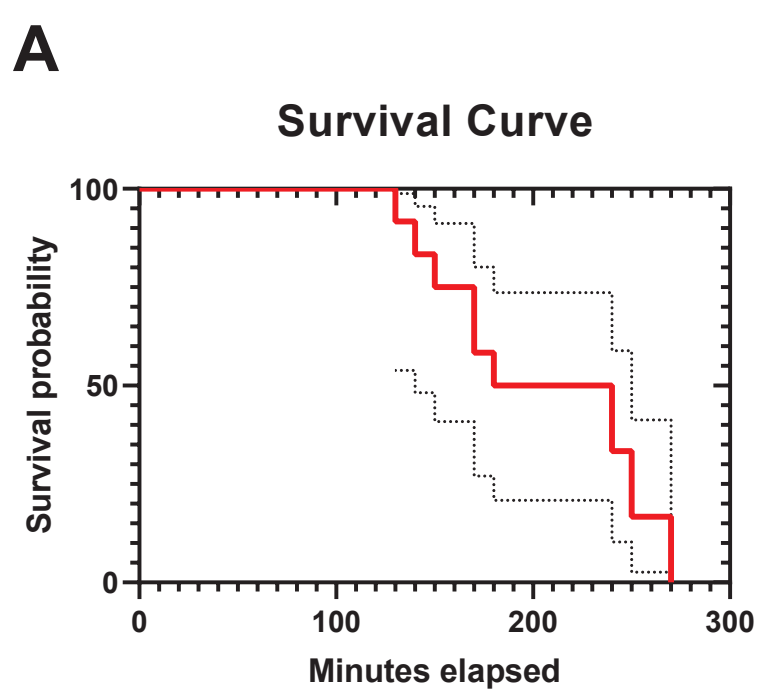

**B**

| Sample | Survival time (min) |
|--------|---------------------|
| FH1    | 130                 |
| FH2    | 140                 |
| FH3    | 150                 |
| FH4    | 170                 |
| FH5    | 170                 |
| FH6    | 180                 |
| FH7    | 240                 |
| FH8    | 240                 |
| FH9    | 250                 |
| FH10   | 250                 |
| FH11   | 270                 |
| FH12   | 270                 |

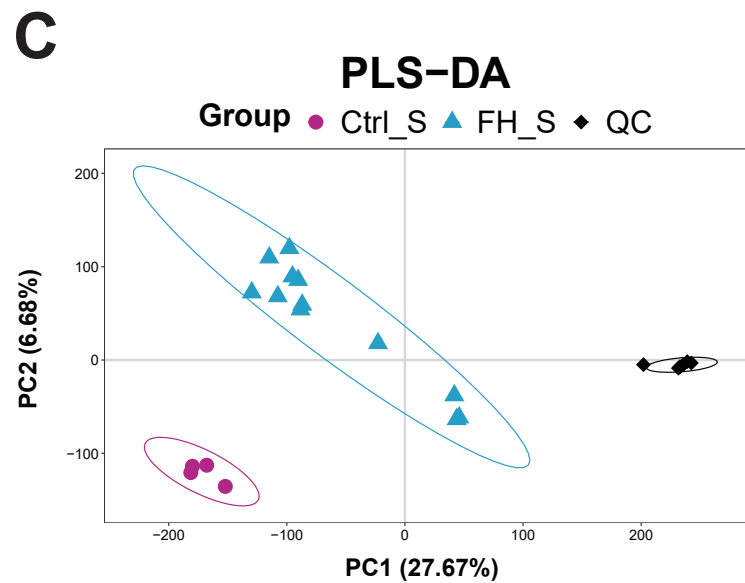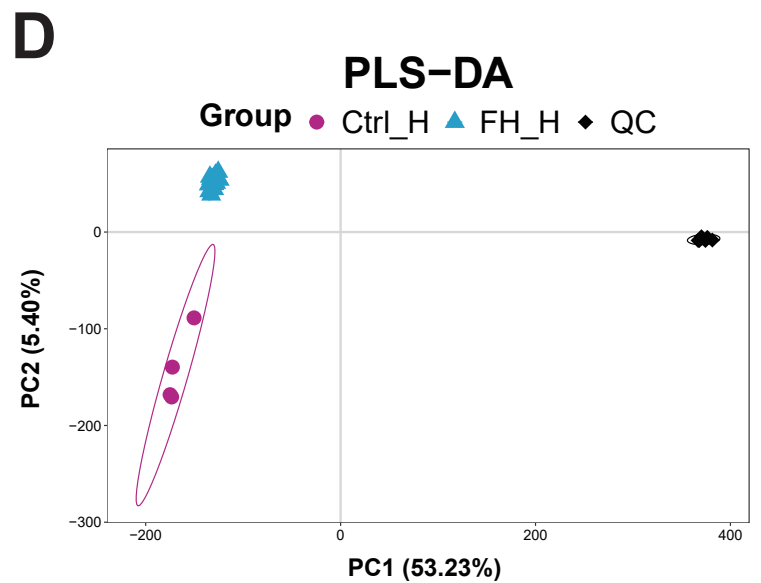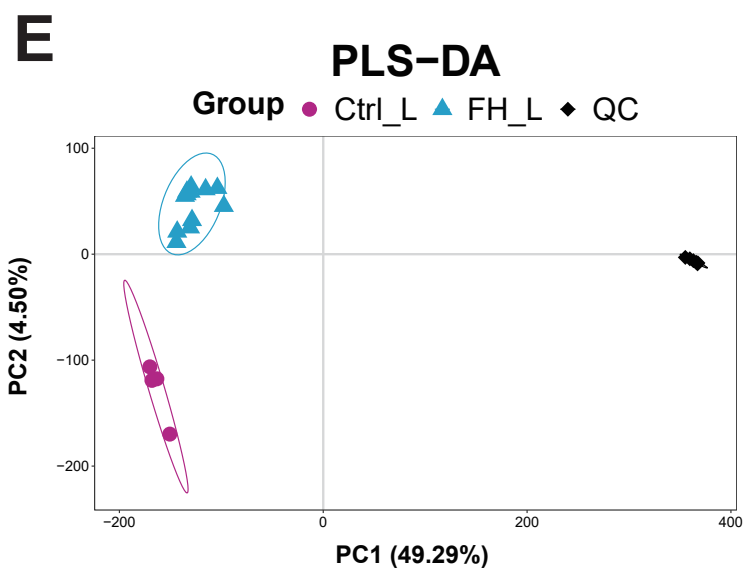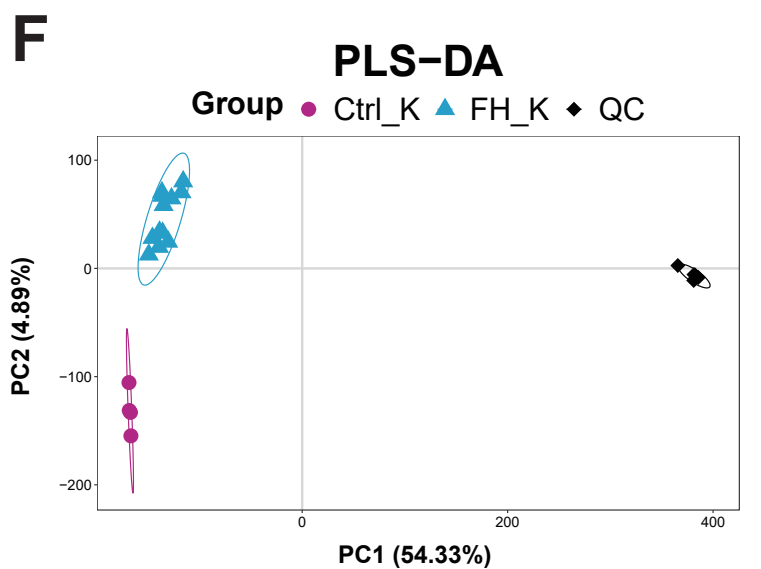

Supplement: Supplementary file 1 [file DataSheet1.zip › Supplemental Fig.1.pdf]
